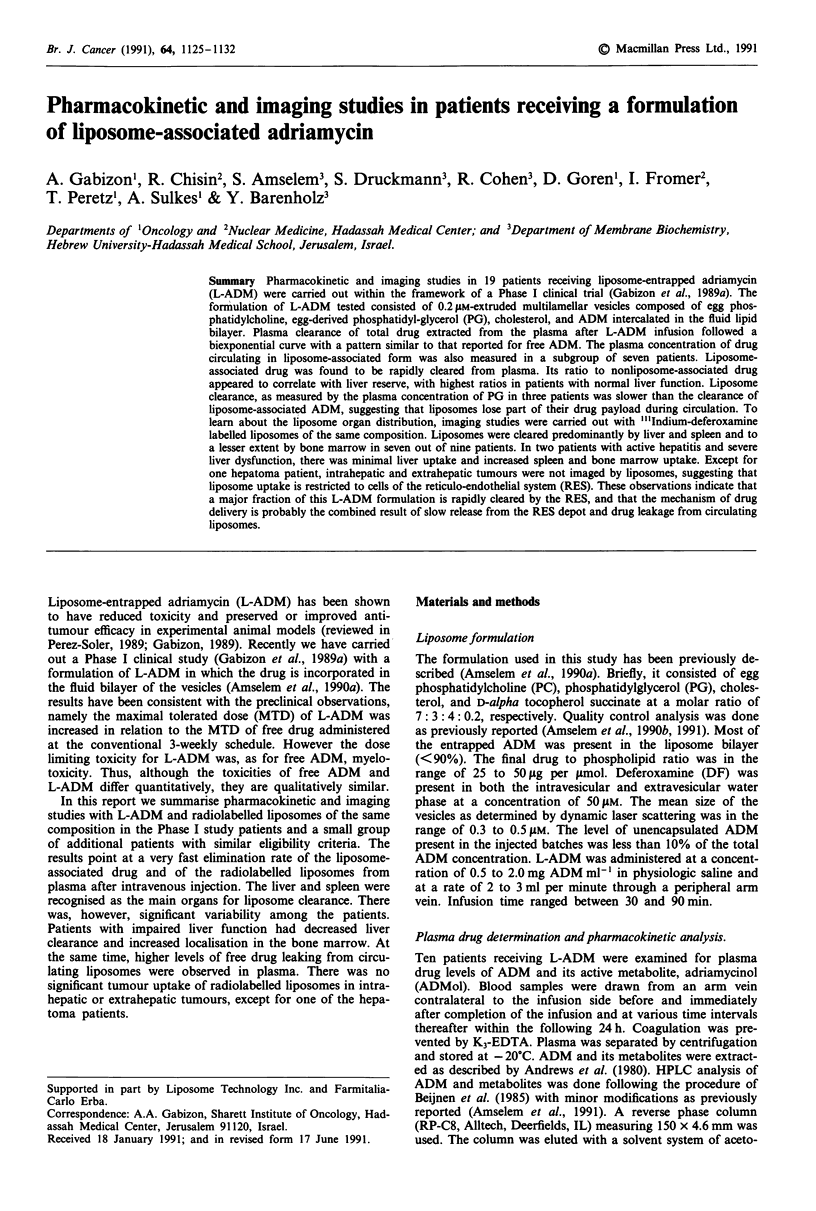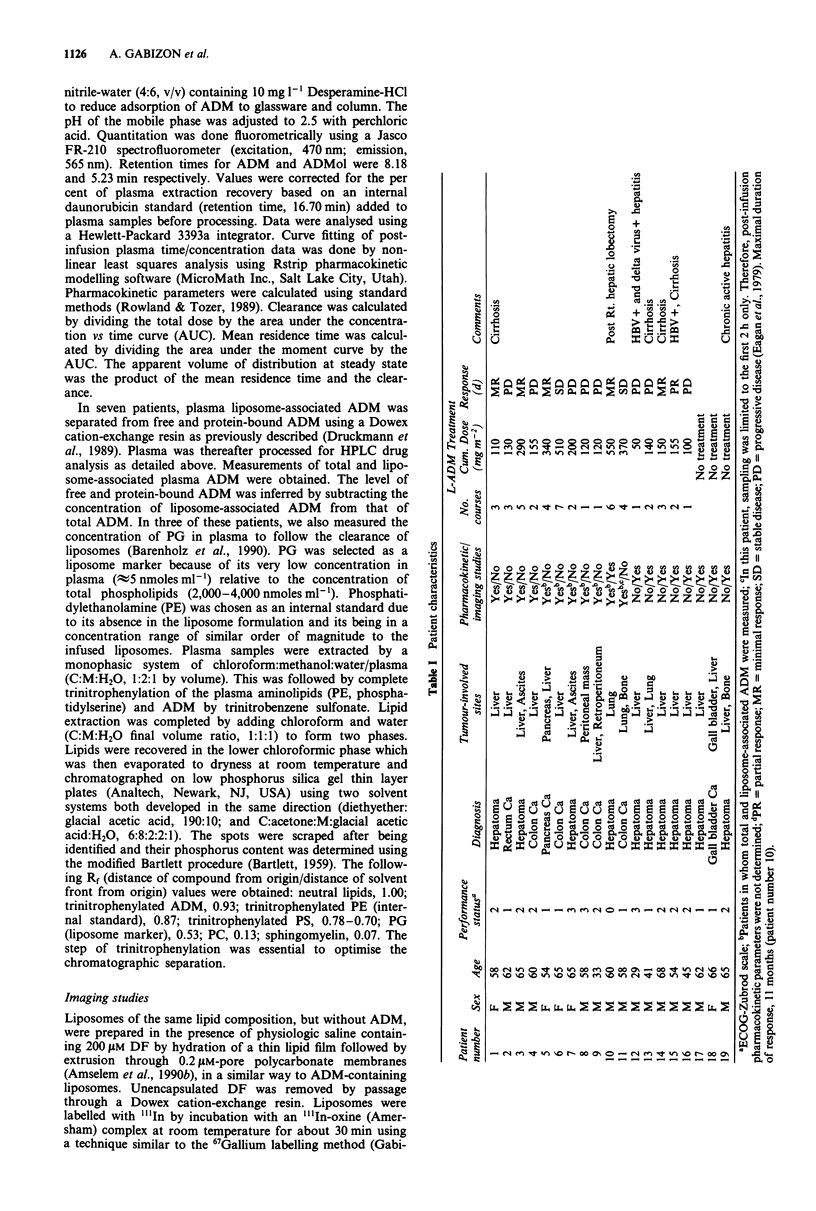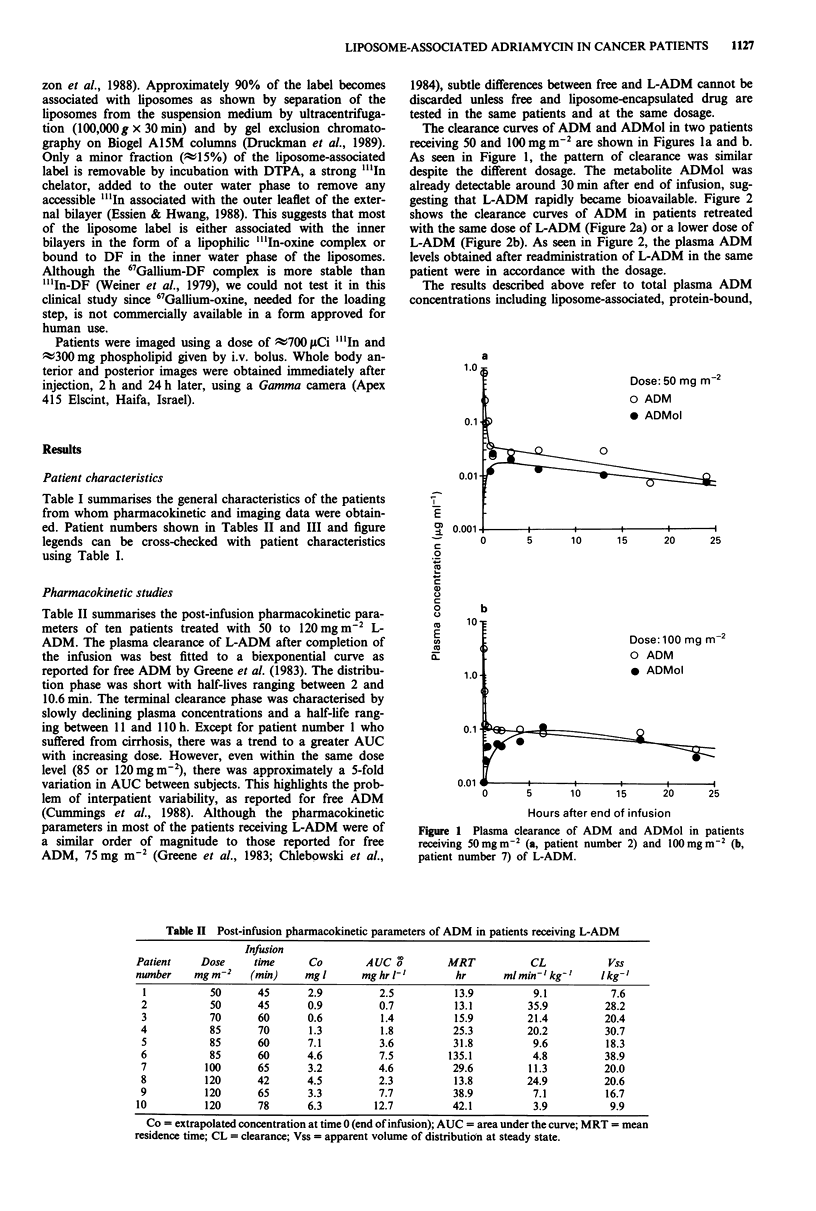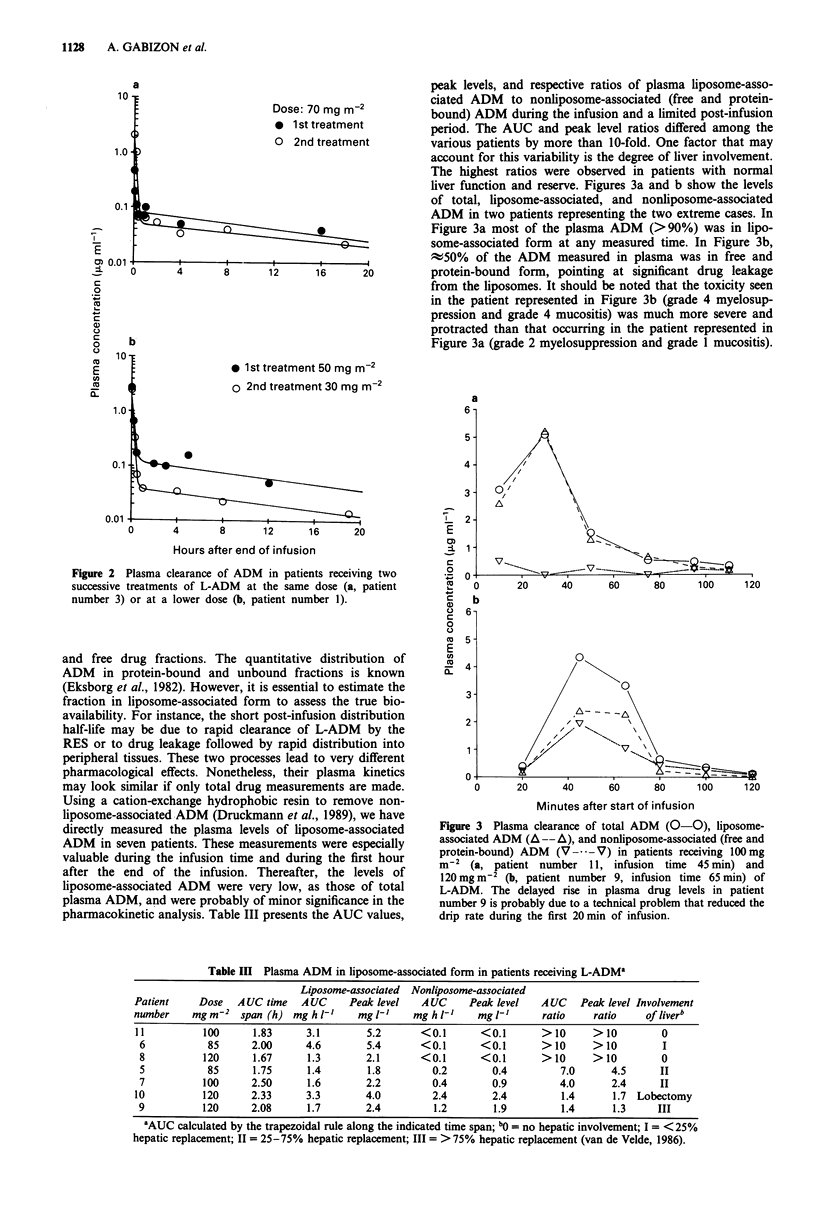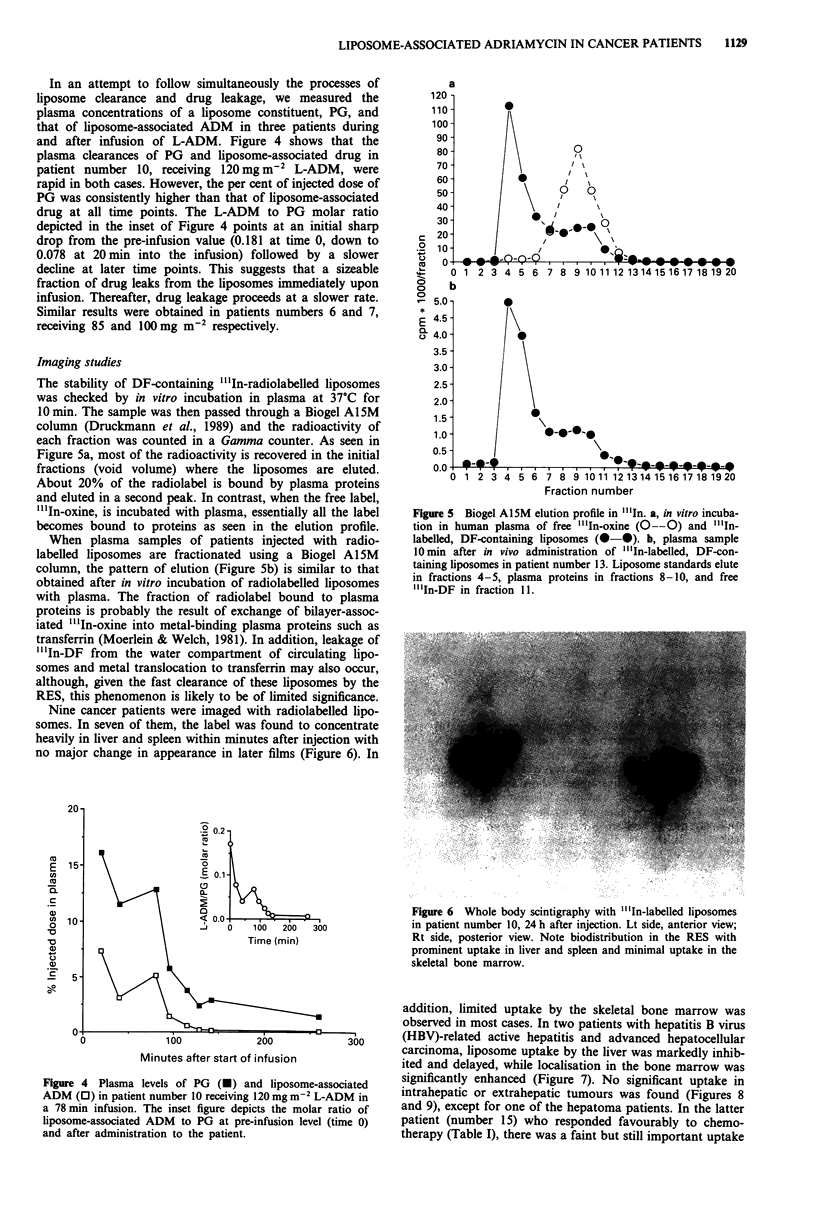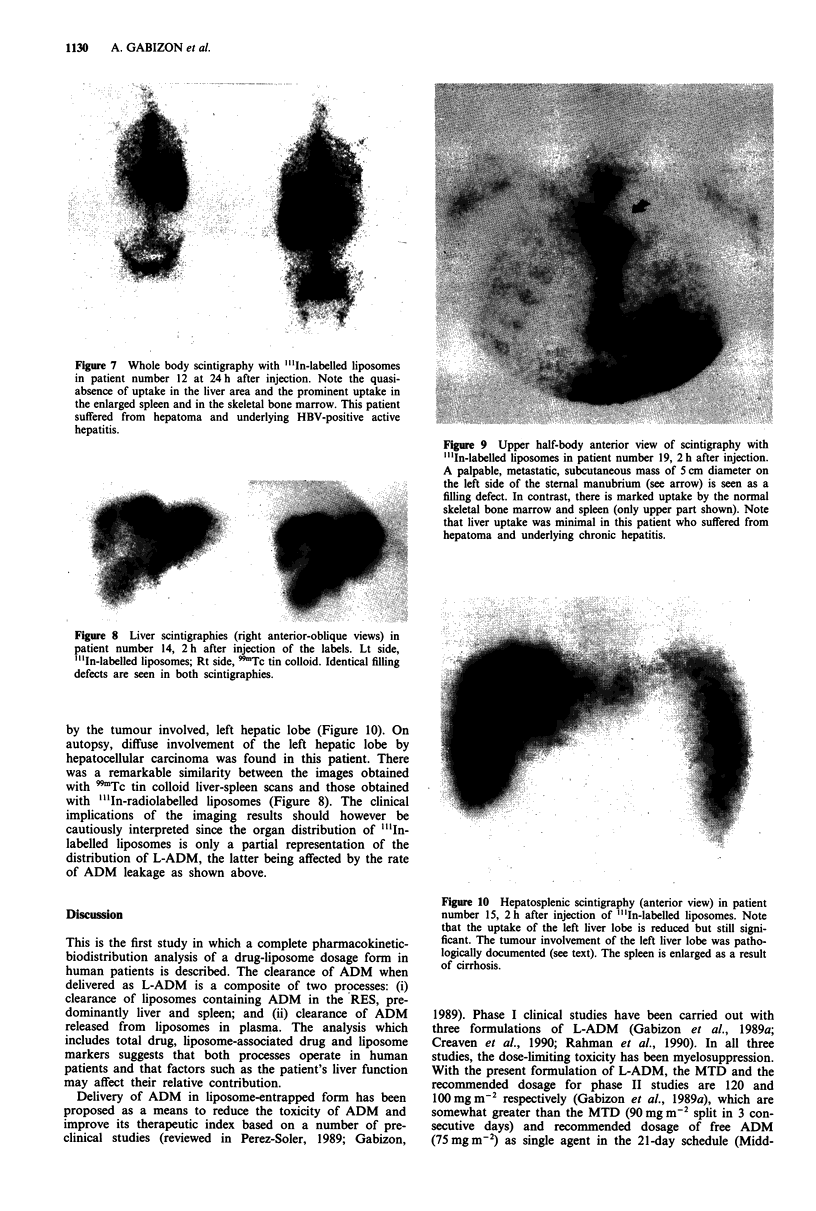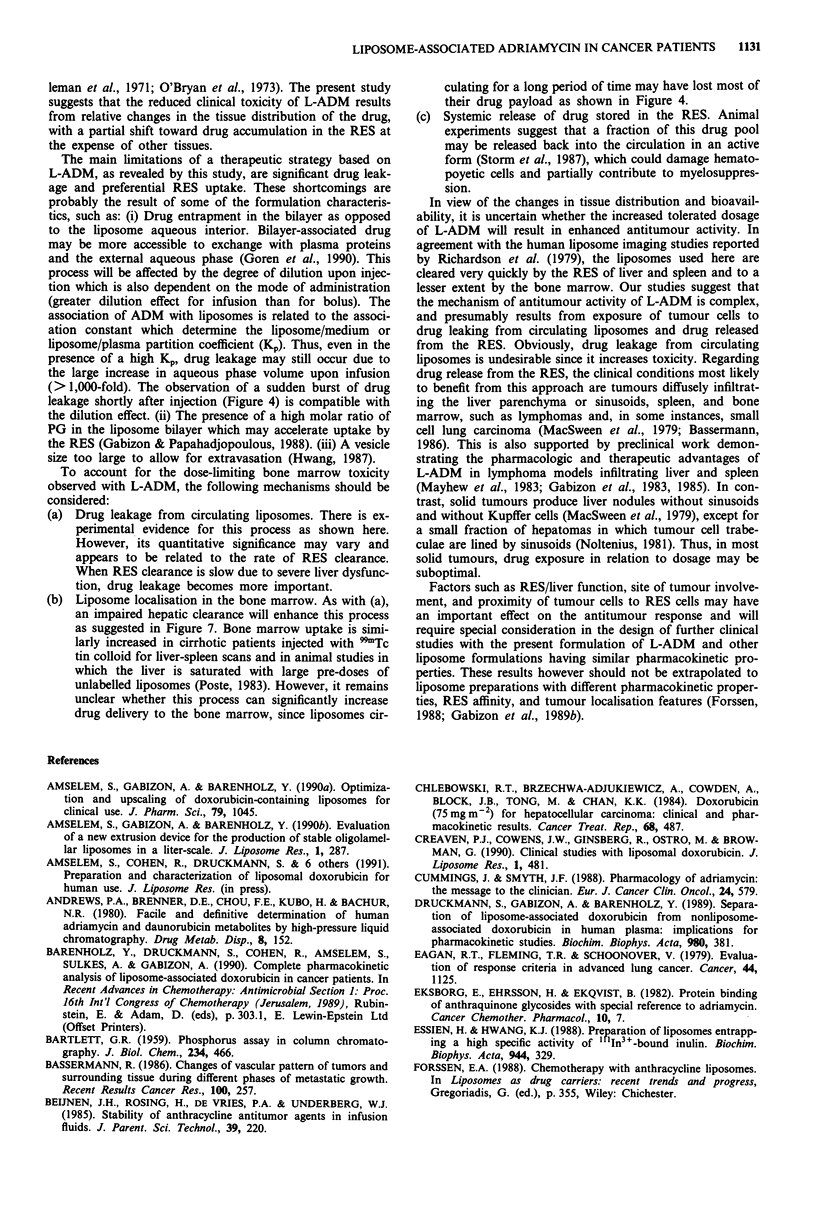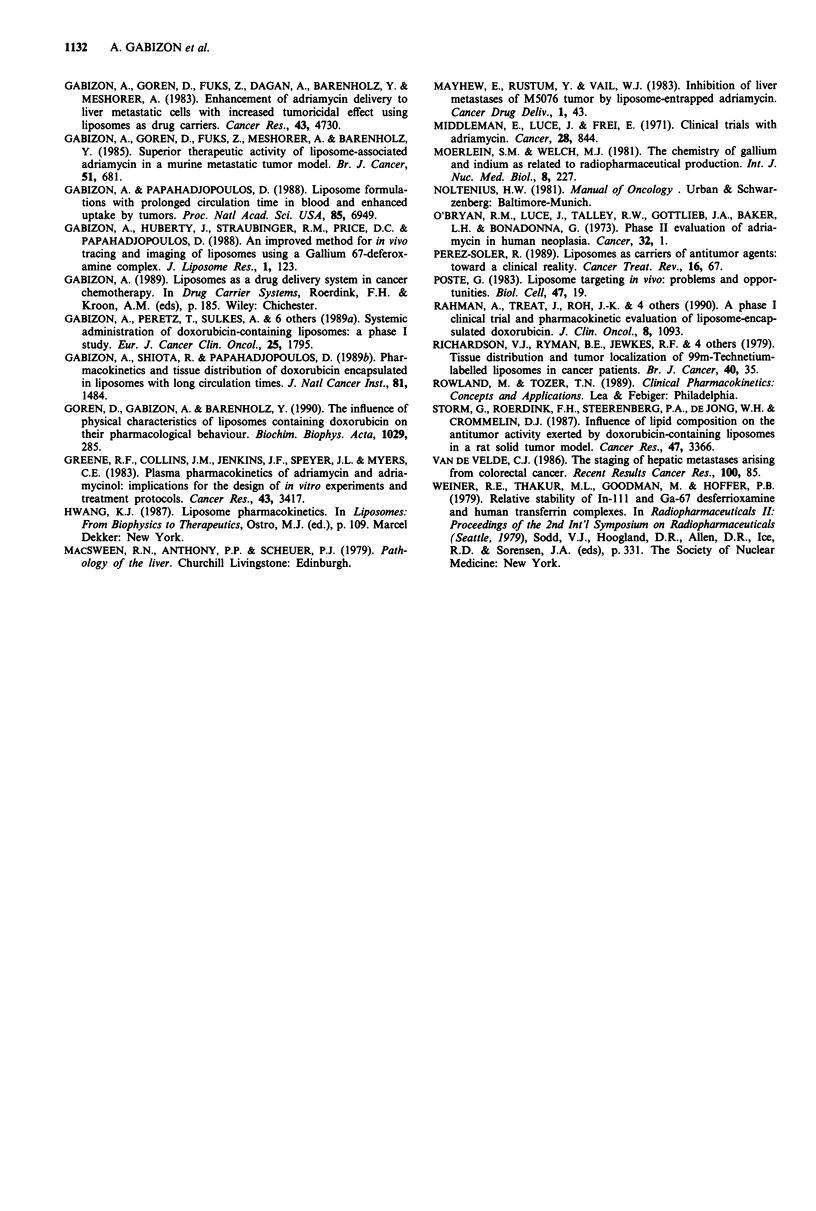# Pharmacokinetic and imaging studies in patients receiving a formulation of liposome-associated adriamycin.

**DOI:** 10.1038/bjc.1991.476

**Published:** 1991-12

**Authors:** A. Gabizon, R. Chisin, S. Amselem, S. Druckmann, R. Cohen, D. Goren, I. Fromer, T. Peretz, A. Sulkes, Y. Barenholz

**Affiliations:** Department of Oncology, Hadassah Medical Center, Jerusalem, Israel.

## Abstract

**Images:**


Br. J. Cancer (1991), 64, 1125–1132 © Macmillan Press Ltd., 1991

### Pharmacokinetic and imaging studies in patients receiving a formulation of liposome-associated adriamycin

A. Gabizon[1], R. Chisin[2], S. Amselem[3], S. Druckmann[3], R. Cohen[3], D. Goren[1], I. Fromer[2], T. Peretz[1], A. Sulkes[1] & Y. Barenholz[3]

*Departments of [1]Oncology and [2]Nuclear Medicine, Hadassah Medical Center; and [3]Department of Membrane Biochemistry, Hebrew University-Hadassah Medical School, Jerusalem, Israel.*

**Summary** Pharmacokinetic and imaging studies in 19 patients receiving liposome-entrapped adriamycin (L-ADM) were carried out within the framework of a Phase I clinical trial (Gabizon *et al.*, 1989*a*). The formulation of L-ADM tested consisted of 0.2 μM-extruded multilamellar vesicles composed of egg phosphatidylcholine, egg-derived phosphatidyl-glycerol (PG), cholesterol, and ADM intercalated in the fluid lipid bilayer. Plasma clearance of total drug extracted from the plasma after L-ADM infusion followed a biexponential curve with a pattern similar to that reported for free ADM. The plasma concentration of drug circulating in liposome-associated form was also measured in a subgroup of seven patients. Liposome-associated drug was found to be rapidly cleared from plasma. Its ratio to nonliposome-associated drug appeared to correlate with liver reserve, with highest ratios in patients with normal liver function. Liposome clearance, as measured by the plasma concentration of PG in three patients was slower than the clearance of liposome-associated ADM, suggesting that liposomes lose part of their drug payload during circulation. To learn about the liposome organ distribution, imaging studies were carried out with $^{111}$Indium-deferoxamine labelled liposomes of the same composition. Liposomes were cleared predominantly by liver and spleen and to a lesser extent by bone marrow in seven out of nine patients. In two patients with active hepatitis and severe liver dysfunction, there was minimal liver uptake and increased spleen and bone marrow uptake. Except for one hepatoma patient, intrahepatic and extrahepatic tumours were not imaged by liposomes, suggesting that liposome uptake is restricted to cells of the reticulo-endothelial system (RES). These observations indicate that a major fraction of this L-ADM formulation is rapidly cleared by the RES, and that the mechanism of drug delivery is probably the combined result of slow release from the RES depot and drug leakage from circulating liposomes.

Liposome-entrapped adriamycin (L-ADM) has been shown to have reduced toxicity and preserved or improved antitumour efficacy in experimental animal models (reviewed in Perez-Soler, 1989; Gabizon, 1989). Recently we have carried out a Phase I clinical study (Gabizon *et al.*, 1989*a*) with a formulation of L-ADM in which the drug is incorporated in the fluid bilayer of the vesicles (Amselem *et al.*, 1990*a*). The results have been consistent with the preclinical observations, namely the maximal tolerated dose (MTD) of L-ADM was increased in relation to the MTD of free drug administered at the conventional 3-weekly schedule. However the dose limiting toxicity for L-ADM was, as for free ADM, myelotoxicity. Thus, although the toxicities of free ADM and L-ADM differ quantitatively, they are qualitatively similar.

In this report we summarise pharmacokinetic and imaging studies with L-ADM and radiolabelled liposomes of the same composition in the Phase I study patients and a small group of additional patients with similar eligibility criteria. The results point at a very fast elimination rate of the liposome-associated drug and of the radiolabelled liposomes from plasma after intravenous injection. The liver and spleen were recognised as the main organs for liposome clearance. There was, however, significant variability among the patients. Patients with impaired liver function had decreased liver clearance and increased localisation in the bone marrow. At the same time, higher levels of free drug leaking from circulating liposomes were observed in plasma. There was no significant tumour uptake of radiolabelled liposomes in intrahepatic or extrahepatic tumours, except for one of the hepatoma patients.

Supported in part by Liposome Technology Inc. and Farmitalia-Carlo Erba.
Correspondence: A.A. Gabizon, Sharett Institute of Oncology, Hadassah Medical Center, Jerusalem 91120, Israel.
Received 18 January 1991; and in revised form 17 June 1991.

### Materials and methods

### *Liposome formulation*

The formulation used in this study has been previously described (Amselem *et al.*, 1990*a*). Briefly, it consisted of egg phosphatidylcholine (PC), phosphatidylglycerol (PG), cholesterol, and D-*alpha* tocopherol succinate at a molar ratio of 7 : 3 : 4 : 0.2, respectively. Quality control analysis was done as previously reported (Amselem *et al.*, 1990*b*, 1991). Most of the entrapped ADM was present in the liposome bilayer (<90%). The final drug to phospholipid ratio was in the range of 25 to 50 μg per μmol. Deferoxamine (DF) was present in both the intravesicular and extravesicular water phase at a concentration of 50 μM. The mean size of the vesicles as determined by dynamic laser scattering was in the range of 0.3 to 0.5 μM. The level of unencapsulated ADM present in the injected batches was less than 10% of the total ADM concentration. L-ADM was administered at a concentration of 0.5 to 2.0 mg ADM $ml^{-1}$ in physiologic saline and at a rate of 2 to 3 ml per minute through a peripheral arm vein. Infusion time ranged between 30 and 90 min.

### *Plasma drug determination and pharmacokinetic analysis.*

Ten patients receiving L-ADM were examined for plasma drug levels of ADM and its active metabolite, adriamycinol (ADMol). Blood samples were drawn from an arm vein contralateral to the infusion side before and immediately after completion of the infusion and at various time intervals thereafter within the following 24 h. Coagulation was prevented by $K_3$-EDTA. Plasma was separated by centrifugation and stored at −20°C. ADM and its metabolites were extracted as described by Andrews *et al.* (1980). HPLC analysis of ADM and metabolites was done following the procedure of Beijnen *et al.* (1985) with minor modifications as previously reported (Amselem *et al.*, 1991). A reverse phase column (RP-C8, Alltech, Deerfields, IL) measuring 150 × 4.6 mm was used. The column was eluted with a solvent system of acetonitrile-water (4:6, v/v) containing 10 mg $l^{-1}$ Desperamine-HCl to reduce adsorption of ADM to glassware and column. The pH of the mobile phase was adjusted to 2.5 with perchloric acid. Quantitation was done fluorometrically using a Jasco FR-210 spectrofluorometer (excitation, 470 nm; emission, 565 nm). Retention times for ADM and ADMol were 8.18 and 5.23 min respectively. Values were corrected for the per cent of plasma extraction recovery based on an internal daunorubicin standard (retention time, 16.70 min) added to plasma samples before processing. Data were analysed using a Hewlett-Packard 3393a integrator. Curve fitting of post-infusion plasma time/concentration data was done by non-linear least squares analysis using Rstrip pharmacokinetic modelling software (MicroMath Inc., Salt Lake City, Utah). Pharmacokinetic parameters were calculated using standard methods (Rowland & Tozer, 1989). Clearance was calculated by dividing the total dose by the area under the concentration *vs* time curve (AUC). Mean residence time was calculated by dividing the area under the moment curve by the AUC. The apparent volume of distribution at steady state was the product of the mean residence time and the clearance.

In seven patients, plasma liposome-associated ADM was separated from free and protein-bound ADM using a Dowex cation-exchange resin as previously described (Druckmann *et al.*, 1989). Plasma was thereafter processed for HPLC drug analysis as detailed above. Measurements of total and liposome-associated plasma ADM were obtained. The level of free and protein-bound ADM was inferred by subtracting the concentration of liposome-associated ADM from that of total ADM. In three of these patients, we also measured the concentration of PG in plasma to follow the clearance of liposomes (Barenholz *et al.*, 1990). PG was selected as a liposome marker because of its very low concentration in plasma (≈5 nmoles $ml^{-1}$) relative to the concentration of total phospholipids (2,000–4,000 nmoles $ml^{-1}$). Phosphatidylethanolamine (PE) was chosen as an internal standard due to its absence in the liposome formulation and its being in a concentration range of similar order of magnitude to the infused liposomes. Plasma samples were extracted by a monophasic system of chloroform:methanol:water/plasma (C:M:$H_2O$, 1:2:1 by volume). This was followed by complete trinitrophenylation of the plasma aminolipids (PE, phosphatidylserine) and ADM by trinitrobenzene sulfonate. Lipid extraction was completed by adding chloroform and water (C:M:$H_2O$ final volume ratio, 1:1:1) to form two phases. Lipids were recovered in the lower chloroformic phase which was then evaporated to dryness at room temperature and chromatographed on low phosphorus silica gel thin layer plates (Analtech, Newark, NJ, USA) using two solvent systems both developed in the same direction (diethyether: glacial acetic acid, 190:10; and C:acetone:M:glacial acetic acid:$H_2O$, 6:8:2:2:1). The spots were scraped after being identified and their phosphorus content was determined using the modified Bartlett procedure (Bartlett, 1959). The following $R_f$ (distance of compound from origin/distance of solvent front from origin) values were obtained: neutral lipids, 1.00; trinitrophenylated ADM, 0.93; trinitrophenylated PE (internal standard), 0.87; trinitrophenylated PS, 0.78–0.70; PG (liposome marker), 0.53; PC, 0.13; sphingomyelin, 0.07. The step of trinitrophenylation was essential to optimise the chromatographic separation.

*Imaging studies*

Liposomes of the same lipid composition, but without ADM, were prepared in the presence of physiologic saline containing 200 μM DF by hydration of a thin lipid film followed by extrusion through 0.2 μM-pore polycarbonate membranes (Amselem *et al.*, 1990*b*), in a similar way to ADM-containing liposomes. Unencapsulated DF was removed by passage through a Dowex cation-exchange resin. Liposomes were labelled with $^{111}$In by incubation with an $^{111}$In-oxine (Amersham) complex at room temperature for about 30 min using a technique similar to the $^{67}$Gallium labelling method (Gabi-

**Table I** Patient characteristics

| Patient number | Sex | Age | Performance status[a] | Diagnosis | Tumour-involved sites | Pharmacokinetic/ imaging studies | L-ADM Treatment No. courses | Cum. Dose ($mg\ m^{-2}$) | Response (d) | Comments |
|---|---|---|---|---|---|---|---|---|---|---|
| 1 | F | 58 | 2 | Hepatoma | Liver | Yes/No | 3 | 110 | MR | Cirrhosis |
| 2 | M | 62 | 1 | Rectum Ca | Liver | Yes/No | 3 | 130 | PD | |
| 3 | M | 65 | 2 | Hepatoma | Liver, Ascites | Yes/No | 5 | 290 | MR | |
| 4 | M | 60 | 2 | Colon Ca | Liver | Yes/No | 2 | 155 | PD | |
| 5 | F | 54 | 1 | Pancreas Ca | Pancreas, Liver | Yes[b]/No | 4 | 340 | MR | |
| 6 | F | 65 | 1 | Colon Ca | Liver | Yes[b]/No | 7 | 510 | SD | |
| 7 | F | 65 | 3 | Hepatoma | Liver, Ascites | Yes[b]/No | 2 | 200 | PD | |
| 8 | M | 58 | 3 | Colon Ca | Peritoneal mass | Yes[b]/No | 1 | 120 | PD | |
| 9 | M | 33 | 2 | Colon Ca | Liver, Retroperitoneum | Yes[b]/No | 1 | 120 | PD | |
| 10 | M | 60 | 0 | Hepatoma | Lung | Yes[b]/Yes | 6 | 550 | MR | Post Rt. hepatic lobectomy |
| 11 | M | 58 | 1 | Colon Ca | Lung, Bone | Yes[b,c]/No | 4 | 370 | SD | |
| 12 | M | 29 | 3 | Hepatoma | Liver | No/Yes | 1 | 50 | PD | HBV+ and delta virus+ hepatitis |
| 13 | M | 41 | 1 | Hepatoma | Liver, Lung | No/Yes | 2 | 140 | PD | Cirrhosis |
| 14 | M | 68 | 2 | Hepatoma | Liver | No/Yes | 3 | 150 | MR | Cirrhosis |
| 15 | M | 54 | 2 | Hepatoma | Liver | No/Yes | 2 | 155 | PR | HBV+, Cirrhosis |
| 16 | M | 45 | 2 | Hepatoma | Liver | No/Yes | 1 | 100 | PD | |
| 17 | M | 62 | 1 | Hepatoma | Liver | No/Yes | | No treatment | | |
| 18 | F | 66 | 1 | Gall bladder Ca | Gall bladder, Liver | No/Yes | | No treatment | | |
| 19 | M | 65 | 2 | Hepatoma | Liver, Bone | No/Yes | | No treatment | | Chronic active hepatitis |

[a]ECOG-Zubrod scale; [b]Patients in whom total and liposome-associated ADM were measured; [c]In this patient, sampling was limited to the first 2 h only. Therefore, post-infusion pharmacokinetic parameters were not determined; [d]PR = partial response; MR = minimal response; SD = stable disease; PD = progressive disease (Eagan *et al.*, 1979). Maximal duration of response, 11 months (patient number 10).

zon *et al.*, 1988). Approximately 90% of the label becomes associated with liposomes as shown by separation of the liposomes from the suspension medium by ultracentrifugation (100,000 *g* × 30 min) and by gel exclusion chromatography on Biogel A15M columns (Druckman *et al.*, 1989). Only a minor fraction (≈15%) of the liposome-associated label is removable by incubation with DTPA, a strong $^{111}$In chelator, added to the outer water phase to remove any accessible $^{111}$In associated with the outer leaflet of the external bilayer (Essien & Hwang, 1988). This suggests that most of the liposome label is either associated with the inner bilayers in the form of a lipophilic $^{111}$In-oxine complex or bound to DF in the inner water phase of the liposomes. Although the $^{67}$Gallium-DF complex is more stable than $^{111}$In-DF (Weiner *et al.*, 1979), we could not test it in this clinical study since $^{67}$Gallium-oxine, needed for the loading step, is not commercially available in a form approved for human use.

Patients were imaged using a dose of ≈700 μCi $^{111}$In and ≈300 mg phospholipid given by i.v. bolus. Whole body anterior and posterior images were obtained immediately after injection, 2 h and 24 h later, using a *Gamma* camera (Apex 415 Elscint, Haifa, Israel).

### Results

### *Patient characteristics*

Table I summarises the general characteristics of the patients from whom pharmacokinetic and imaging data were obtained. Patient numbers shown in Tables II and III and figure legends can be cross-checked with patient characteristics using Table I.

### *Pharmacokinetic studies*

Table II summarises the post-infusion pharmacokinetic parameters of ten patients treated with 50 to 120 mg m$^{-2}$ L-ADM. The plasma clearance of L-ADM after completion of the infusion was best fitted to a biexponential curve as reported for free ADM by Greene *et al.* (1983). The distribution phase was short with half-lives ranging between 2 and 10.6 min. The terminal clearance phase was characterised by slowly declining plasma concentrations and a half-life ranging between 11 and 110 h. Except for patient number 1 who suffered from cirrhosis, there was a trend to a greater AUC with increasing dose. However, even within the same dose level (85 or 120 mg m$^{-2}$), there was approximately a 5-fold variation in AUC between subjects. This highlights the problem of interpatient variability, as reported for free ADM (Cummings *et al.*, 1988). Although the pharmacokinetic parameters in most of the patients receiving L-ADM were of a similar order of magnitude to those reported for free ADM, 75 mg m$^{-2}$ (Greene *et al.*, 1983; Chlebowski *et al.*, 1984), subtle differences between free and L-ADM cannot be discarded unless free and liposome-encapsulated drug are tested in the same patients and at the same dosage.

The clearance curves of ADM and ADMol in two patients receiving 50 and 100 mg m$^{-2}$ are shown in Figures 1a and b. As seen in Figure 1, the pattern of clearance was similar despite the different dosage. The metabolite ADMol was already detectable around 30 min after end of infusion, suggesting that L-ADM rapidly became bioavailable. Figure 2 shows the clearance curves of ADM in patients retreated with the same dose of L-ADM (Figure 2a) or a lower dose of L-ADM (Figure 2b). As seen in Figure 2, the plasma ADM levels obtained after readministration of L-ADM in the same patient were in accordance with the dosage.

The results described above refer to total plasma ADM concentrations including liposome-associated, protein-bound,

**Figure 1** Plasma clearance of ADM and ADMol in patients receiving 50 mg m$^{-2}$ (**a**, patient number 2) and 100 mg m$^{-2}$ (**b**, patient number 7) of L-ADM.

**Table II** Post-infusion pharmacokinetic parameters of ADM in patients receiving L-ADM

| *Patient number* | *Dose mg m$^{-2}$* | *Infusion time (min)* | *Co mg l* | *AUC $_0^\infty$ mg hr l$^{-1}$* | *MRT hr* | *CL ml min$^{-1}$ kg$^{-1}$* | *Vss l kg$^{-1}$* |
|---|---|---|---|---|---|---|---|
| 1 | 50 | 45 | 2.9 | 2.5 | 13.9 | 9.1 | 7.6 |
| 2 | 50 | 45 | 0.9 | 0.7 | 13.1 | 35.9 | 28.2 |
| 3 | 70 | 60 | 0.6 | 1.4 | 15.9 | 21.4 | 20.4 |
| 4 | 85 | 70 | 1.3 | 1.8 | 25.3 | 20.2 | 30.7 |
| 5 | 85 | 60 | 7.1 | 3.6 | 31.8 | 9.6 | 18.3 |
| 6 | 85 | 60 | 4.6 | 7.5 | 135.1 | 4.8 | 38.9 |
| 7 | 100 | 65 | 3.2 | 4.6 | 29.6 | 11.3 | 20.0 |
| 8 | 120 | 42 | 4.5 | 2.3 | 13.8 | 24.9 | 20.6 |
| 9 | 120 | 65 | 3.3 | 7.7 | 38.9 | 7.1 | 16.7 |
| 10 | 120 | 78 | 6.3 | 12.7 | 42.1 | 3.9 | 9.9 |

Co = extrapolated concentration at time 0 (end of infusion); AUC = area under the curve; MRT = mean residence time; CL = clearance; Vss = apparent volume of distribution at steady state.

**Figure 2** Plasma clearance of ADM in patients receiving two successive treatments of L-ADM at the same dose (**a**, patient number 3) or at a lower dose (**b**, patient number 1).

and free drug fractions. The quantitative distribution of ADM in protein-bound and unbound fractions is known (Eksborg *et al.*, 1982). However, it is essential to estimate the fraction in liposome-associated form to assess the true bioavailability. For instance, the short post-infusion distribution half-life may be due to rapid clearance of L-ADM by the RES or to drug leakage followed by rapid distribution into peripheral tissues. These two processes lead to very different pharmacological effects. Nonetheless, their plasma kinetics may look similar if only total drug measurements are made. Using a cation-exchange hydrophobic resin to remove nonliposome-associated ADM (Druckmann *et al.*, 1989), we have directly measured the plasma levels of liposome-associated ADM in seven patients. These measurements were especially valuable during the infusion time and during the first hour after the end of the infusion. Thereafter, the levels of liposome-associated ADM were very low, as those of total plasma ADM, and were probably of minor significance in the pharmacokinetic analysis. Table III presents the AUC values, peak levels, and respective ratios of plasma liposome-associated ADM to nonliposome-associated (free and protein-bound) ADM during the infusion and a limited post-infusion period. The AUC and peak level ratios differed among the various patients by more than 10-fold. One factor that may account for this variability is the degree of liver involvement. The highest ratios were observed in patients with normal liver function and reserve. Figures 3a and b show the levels of total, liposome-associated, and nonliposome-associated ADM in two patients representing the two extreme cases. In Figure 3a most of the plasma ADM (>90%) was in liposome-associated form at any measured time. In Figure 3b, ≈50% of the ADM measured in plasma was in free and protein-bound form, pointing at significant drug leakage from the liposomes. It should be noted that the toxicity seen in the patient represented in Figure 3b (grade 4 myelosuppression and grade 4 mucositis) was much more severe and protracted than that occurring in the patient represented in Figure 3a (grade 2 myelosuppression and grade 1 mucositis).

**Figure 3** Plasma clearance of total ADM (O—O), liposome-associated ADM (Δ -- Δ), and nonliposome-associated (free and protein-bound) ADM (∇-··-∇) in patients receiving 100 mg m$^{-2}$ (**a**, patient number 11, infusion time 45 min) and 120 mg m$^{-2}$ (**b**, patient number 9, infusion time 65 min) of L-ADM. The delayed rise in plasma drug levels in patient number 9 is probably due to a technical problem that reduced the drip rate during the first 20 min of infusion.

**Table III** Plasma ADM in liposome-associated form in patients receiving L-ADM[a]

| | | | Liposome-associated | | Nonliposome-associated | | | | |
|---|---|---|---|---|---|---|---|---|---|
| *Patient number* | *Dose mg m$^{-2}$* | *AUC time span (h)* | *AUC mg h l$^{-1}$* | *Peak level mg l$^{-1}$* | *AUC mg h l$^{-1}$* | *Peak level mg l$^{-1}$* | *AUC ratio* | *Peak level ratio* | *Involvement of liver*[b] |
| 11 | 100 | 1.83 | 3.1 | 5.2 | <0.1 | <0.1 | >10 | >10 | 0 |
| 6 | 85 | 2.00 | 4.6 | 5.4 | <0.1 | <0.1 | >10 | >10 | I |
| 8 | 120 | 1.67 | 1.3 | 2.1 | <0.1 | <0.1 | >10 | >10 | 0 |
| 5 | 85 | 1.75 | 1.4 | 1.8 | 0.2 | 0.4 | 7.0 | 4.5 | II |
| 7 | 100 | 2.50 | 1.6 | 2.2 | 0.4 | 0.9 | 4.0 | 2.4 | II |
| 10 | 120 | 2.33 | 3.3 | 4.0 | 2.4 | 2.4 | 1.4 | 1.7 | Lobectomy |
| 9 | 120 | 2.08 | 1.7 | 2.4 | 1.2 | 1.9 | 1.4 | 1.3 | III |

[a]AUC calculated by the trapezoidal rule along the indicated time span; [b]0 = no hepatic involvement; I = <25% hepatic replacement; II = 25–75% hepatic replacement; III = >75% hepatic replacement (van de Velde, 1986).

In an attempt to follow simultaneously the processes of liposome clearance and drug leakage, we measured the plasma concentrations of a liposome constituent, PG, and that of liposome-associated ADM in three patients during and after infusion of L-ADM. Figure 4 shows that the plasma clearances of PG and liposome-associated drug in patient number 10, receiving 120 mg m$^{-2}$ L-ADM, were rapid in both cases. However, the per cent of injected dose of PG was consistently higher than that of liposome-associated drug at all time points. The L-ADM to PG molar ratio depicted in the inset of Figure 4 points at an initial sharp drop from the pre-infusion value (0.181 at time 0, down to 0.078 at 20 min into the infusion) followed by a slower decline at later time points. This suggests that a sizeable fraction of drug leaks from the liposomes immediately upon infusion. Thereafter, drug leakage proceeds at a slower rate. Similar results were obtained in patients numbers 6 and 7, receiving 85 and 100 mg m$^{-2}$ respectively.

*Imaging studies*

The stability of DF-containing $^{111}$In-radiolabelled liposomes was checked by *in vitro* incubation in plasma at 37°C for 10 min. The sample was then passed through a Biogel A15M column (Druckmann *et al.*, 1989) and the radioactivity of each fraction was counted in a *Gamma* counter. As seen in Figure 5a, most of the radioactivity is recovered in the initial fractions (void volume) where the liposomes are eluted. About 20% of the radiolabel is bound by plasma proteins and eluted in a second peak. In contrast, when the free label, $^{111}$In-oxine, is incubated with plasma, essentially all the label becomes bound to proteins as seen in the elution profile.

When plasma samples of patients injected with radiolabelled liposomes are fractionated using a Biogel A15M column, the pattern of elution (Figure 5b) is similar to that obtained after *in vitro* incubation of radiolabelled liposomes with plasma. The fraction of radiolabel bound to plasma proteins is probably the result of exchange of bilayer-associated $^{111}$In-oxine into metal-binding plasma proteins such as transferrin (Moerlein & Welch, 1981). In addition, leakage of $^{111}$In-DF from the water compartment of circulating liposomes and metal translocation to transferrin may also occur, although, given the fast clearance of these liposomes by the RES, this phenomenon is likely to be of limited significance.

Nine cancer patients were imaged with radiolabelled liposomes. In seven of them, the label was found to concentrate heavily in liver and spleen within minutes after injection with no major change in appearance in later films (Figure 6). In addition, limited uptake by the skeletal bone marrow was observed in most cases. In two patients with hepatitis B virus (HBV)-related active hepatitis and advanced hepatocellular carcinoma, liposome uptake by the liver was markedly inhibited and delayed, while localisation in the bone marrow was significantly enhanced (Figure 7). No significant uptake in intrahepatic or extrahepatic tumours was found (Figures 8 and 9), except for one of the hepatoma patients. In the latter patient (number 15) who responded favourably to chemotherapy (Table I), there was a faint but still important uptake

**Figure 5** Biogel A15M elution profile in $^{111}$In. **a**, *in vitro* incubation in human plasma of free $^{111}$In-oxine (O--O) and $^{111}$In-labelled, DF-containing liposomes (●—●). **b**, plasma sample 10 min after *in vivo* administration of $^{111}$In-labelled, DF-containing liposomes in patient number 13. Liposome standards elute in fractions 4–5, plasma proteins in fractions 8–10, and free $^{111}$In-DF in fraction 11.

**Figure 6** Whole body scintigraphy with $^{111}$In-labelled liposomes in patient number 10, 24 h after injection. Lt side, anterior view; Rt side, posterior view. Note biodistribution in the RES with prominent uptake in liver and spleen and minimal uptake in the skeletal bone marrow.

**Figure 4** Plasma levels of PG (■) and liposome-associated ADM (□) in patient number 10 receiving 120 mg m$^{-2}$ L-ADM in a 78 min infusion. The inset figure depicts the molar ratio of liposome-associated ADM to PG at pre-infusion level (time 0) and after administration to the patient.

**Figure 7** Whole body scintigraphy with $^{111}$In-labelled liposomes in patient number 12 at 24 h after injection. Note the quasi-absence of uptake in the liver area and the prominent uptake in the enlarged spleen and in the skeletal bone marrow. This patient suffered from hepatoma and underlying HBV-positive active hepatitis.

**Figure 8** Liver scintigraphies (right anterior-oblique views) in patient number 14, 2 h after injection of the labels. Lt side, $^{111}$In-labelled liposomes; Rt side, $^{99m}$Tc tin colloid. Identical filling defects are seen in both scintigraphies.

by the tumour involved, left hepatic lobe (Figure 10). On autopsy, diffuse involvement of the left hepatic lobe by hepatocellular carcinoma was found in this patient. There was a remarkable similarity between the images obtained with $^{99m}$Tc tin colloid liver-spleen scans and those obtained with $^{111}$In-radiolabelled liposomes (Figure 8). The clinical implications of the imaging results should however be cautiously interpreted since the organ distribution of $^{111}$In-labelled liposomes is only a partial representation of the distribution of L-ADM, the latter being affected by the rate of ADM leakage as shown above.

### Discussion

This is the first study in which a complete pharmacokinetic-biodistribution analysis of a drug-liposome dosage form in human patients is described. The clearance of ADM when delivered as L-ADM is a composite of two processes: (i) clearance of liposomes containing ADM in the RES, predominantly liver and spleen; and (ii) clearance of ADM released from liposomes in plasma. The analysis which includes total drug, liposome-associated drug and liposome markers suggests that both processes operate in human patients and that factors such as the patient's liver function may affect their relative contribution.

Delivery of ADM in liposome-entrapped form has been proposed as a means to reduce the toxicity of ADM and improve its therapeutic index based on a number of pre-clinical studies (reviewed in Perez-Soler, 1989; Gabizon, 1989). Phase I clinical studies have been carried out with three formulations of L-ADM (Gabizon *et al.*, 1989*a*; Creaven *et al.*, 1990; Rahman *et al.*, 1990). In all three studies, the dose-limiting toxicity has been myelosuppression. With the present formulation of L-ADM, the MTD and the recommended dosage for phase II studies are 120 and 100 mg m$^{-2}$ respectively (Gabizon *et al.*, 1989*a*), which are somewhat greater than the MTD (90 mg m$^{-2}$ split in 3 consecutive days) and recommended dosage of free ADM (75 mg m$^{-2}$) as single agent in the 21-day schedule (Midd-

**Figure 9** Upper half-body anterior view of scintigraphy with $^{111}$In-labelled liposomes in patient number 19, 2 h after injection. A palpable, metastatic, subcutaneous mass of 5 cm diameter on the left side of the sternal manubrium (see arrow) is seen as a filling defect. In contrast, there is marked uptake by the normal skeletal bone marrow and spleen (only upper part shown). Note that liver uptake was minimal in this patient who suffered from hepatoma and underlying chronic hepatitis.

**Figure 10** Hepatosplenic scintigraphy (anterior view) in patient number 15, 2 h after injection of $^{111}$In-labelled liposomes. Note that the uptake of the left liver lobe is reduced but still significant. The tumour involvement of the left liver lobe was pathologically documented (see text). The spleen is enlarged as a result of cirrhosis.

leman *et al.*, 1971; O'Bryan *et al.*, 1973). The present study suggests that the reduced clinical toxicity of L-ADM results from relative changes in the tissue distribution of the drug, with a partial shift toward drug accumulation in the RES at the expense of other tissues.

The main limitations of a therapeutic strategy based on L-ADM, as revealed by this study, are significant drug leakage and preferential RES uptake. These shortcomings are probably the result of some of the formulation characteristics, such as: (i) Drug entrapment in the bilayer as opposed to the liposome aqueous interior. Bilayer-associated drug may be more accessible to exchange with plasma proteins and the external aqueous phase (Goren *et al.*, 1990). This process will be affected by the degree of dilution upon injection which is also dependent on the mode of administration (greater dilution effect for infusion than for bolus). The association of ADM with liposomes is related to the association constant which determine the liposome/medium or liposome/plasma partition coefficient ($K_p$). Thus, even in the presence of a high $K_p$, drug leakage may still occur due to the large increase in aqueous phase volume upon infusion ($>$1,000-fold). The observation of a sudden burst of drug leakage shortly after injection (Figure 4) is compatible with the dilution effect. (ii) The presence of a high molar ratio of PG in the liposome bilayer which may accelerate uptake by the RES (Gabizon & Papahadjopoulous, 1988). (iii) A vesicle size too large to allow for extravasation (Hwang, 1987).

To account for the dose-limiting bone marrow toxicity observed with L-ADM, the following mechanisms should be considered:

(a) Drug leakage from circulating liposomes. There is experimental evidence for this process as shown here. However, its quantitative significance may vary and appears to be related to the rate of RES clearance. When RES clearance is slow due to severe liver dysfunction, drug leakage becomes more important.
(b) Liposome localisation in the bone marrow. As with (a), an impaired hepatic clearance will enhance this process as suggested in Figure 7. Bone marrow uptake is similarly increased in cirrhotic patients injected with $^{99m}Tc$ tin colloid for liver-spleen scans and in animal studies in which the liver is saturated with large pre-doses of unlabelled liposomes (Poste, 1983). However, it remains unclear whether this process can significantly increase drug delivery to the bone marrow, since liposomes circulating for a long period of time may have lost most of their drug payload as shown in Figure 4.
(c) Systemic release of drug stored in the RES. Animal experiments suggest that a fraction of this drug pool may be released back into the circulation in an active form (Storm *et al.*, 1987), which could damage hematopoyetic cells and partially contribute to myelosuppression.

In view of the changes in tissue distribution and bioavailability, it is uncertain whether the increased tolerated dosage of L-ADM will result in enhanced antitumour activity. In agreement with the human liposome imaging studies reported by Richardson *et al.* (1979), the liposomes used here are cleared very quickly by the RES of liver and spleen and to a lesser extent by the bone marrow. Our studies suggest that the mechanism of antitumour activity of L-ADM is complex, and presumably results from exposure of tumour cells to drug leaking from circulating liposomes and drug released from the RES. Obviously, drug leakage from circulating liposomes is undesirable since it increases toxicity. Regarding drug release from the RES, the clinical conditions most likely to benefit from this approach are tumours diffusely infiltrating the liver parenchyma or sinusoids, spleen, and bone marrow, such as lymphomas and, in some instances, small cell lung carcinoma (MacSween *et al.*, 1979; Bassermann, 1986). This is also supported by preclinical work demonstrating the pharmacologic and therapeutic advantages of L-ADM in lymphoma models infiltrating liver and spleen (Mayhew *et al.*, 1983; Gabizon *et al.*, 1983, 1985). In contrast, solid tumours produce liver nodules without sinusoids and without Kupffer cells (MacSween *et al.*, 1979), except for a small fraction of hepatomas in which tumour cell trabeculae are lined by sinusoids (Noltenius, 1981). Thus, in most solid tumours, drug exposure in relation to dosage may be suboptimal.

Factors such as RES/liver function, site of tumour involvement, and proximity of tumour cells to RES cells may have an important effect on the antitumour response and will require special consideration in the design of further clinical studies with the present formulation of L-ADM and other liposome formulations having similar pharmacokinetic properties. These results however should not be extrapolated to liposome preparations with different pharmacokinetic properties, RES affinity, and tumour localisation features (Forssen, 1988; Gabizon *et al.*, 1989*b*).

**References**

AMSELEM, S., GABIZON, A. & BARENHOLZ, Y. (1990*a*). Optimization and upscaling of doxorubicin-containing liposomes for clinical use. *J. Pharm. Sci.*, **79**, 1045.

AMSELEM, S., GABIZON, A. & BARENHOLZ, Y. (1990*b*). Evaluation of a new extrusion device for the production of stable oligolamellar liposomes in a liter-scale. *J. Liposome Res.*, **1**, 287.

AMSELEM, S., COHEN, R., DRUCKMANN, S. & 6 others (1991). Preparation and characterization of liposomal doxorubicin for human use. *J. Liposome Res.* (in press).

ANDREWS, P.A., BRENNER, D.E., CHOU, F.E., KUBO, H. & BACHUR, N.R. (1980). Facile and definitive determination of human adriamycin and daunorubicin metabolites by high-pressure liquid chromatography. *Drug Metab. Disp.*, **8**, 152.

BARENHOLZ, Y., DRUCKMANN, S., COHEN, R., AMSELEM, S., SULKES, A. & GABIZON, A. (1990). Complete pharmacokinetic analysis of liposome-associated doxorubicin in cancer patients. In *Recent Advances in Chemotherapy: Antimicrobial Section 1: Proc. 16th Int'l Congress of Chemotherapy (Jerusalem, 1989)*, Rubinstein, E. & Adam, D. (eds), p. 303.1, E. Lewin-Epstein Ltd (Offset Printers).

BARTLETT, G.R. (1959). Phosphorus assay in column chromatography. *J. Biol. Chem.*, **234**, 466.

BASSERMANN, R. (1986). Changes of vascular pattern of tumors and surrounding tissue during different phases of metastatic growth. *Recent Results Cancer Res.*, **100**, 257.

BEIJNEN, J.H., ROSING, H., DE VRIES, P.A. & UNDERBERG, W.J. (1985). Stability of anthracycline antitumor agents in infusion fluids. *J. Parent. Sci. Technol.*, **39**, 220.

CHLEBOWSKI, R.T., BRZECHWA-ADJUKIEWICZ, A., COWDEN, A., BLOCK, J.B., TONG, M. & CHAN, K.K. (1984). Doxorubicin ($75 \text{ mg m}^{-2}$) for hepatocellular carcinoma: clinical and pharmacokinetic results. *Cancer Treat. Rep.*, **68**, 487.

CREAVEN, P.J., COWENS, J.W., GINSBERG, R., OSTRO, M. & BROWMAN, G. (1990). Clinical studies with liposomal doxorubicin. *J. Liposome Res.*, **1**, 481.

CUMMINGS, J. & SMYTH, J.F. (1988). Pharmacology of adriamycin: the message to the clinician. *Eur. J. Cancer Clin. Oncol.*, **24**, 579.

DRUCKMANN, S., GABIZON, A. & BARENHOLZ, Y. (1989). Separation of liposome-associated doxorubicin from nonliposome-associated doxorubicin in human plasma: implications for pharmacokinetic studies. *Biochim. Biophys. Acta*, **980**, 381.

EAGAN, R.T., FLEMING, T.R. & SCHOONOVER, V. (1979). Evaluation of response criteria in advanced lung cancer. *Cancer*, **44**, 1125.

EKSBORG, E., EHRSSON, H. & EKQVIST, B. (1982). Protein binding of anthraquinone glycosides with special reference to adriamycin. *Cancer Chemother. Pharmacol.*, **10**, 7.

ESSIEN, H. & HWANG, K.J. (1988). Preparation of liposomes entrapping a high specific activity of $^{111}In^{3+}$-bound inulin. *Biochim. Biophys. Acta*, **944**, 329.

FORSSEN, E.A. (1988). Chemotherapy with anthracycline liposomes. In *Liposomes as drug carriers: recent trends and progress*, Gregoriadis, G. (ed.), p. 355, Wiley: Chichester.

GABIZON, A., GOREN, D., FUKS, Z., DAGAN, A., BARENHOLZ, Y. & MESHORER, A. (1983). Enhancement of adriamycin delivery to liver metastatic cells with increased tumoricidal effect using liposomes as drug carriers. *Cancer Res.*, **43**, 4730.

GABIZON, A., GOREN, D., FUKS, Z., MESHORER, A. & BARENHOLZ, Y. (1985). Superior therapeutic activity of liposome-associated adriamycin in a murine metastatic tumor model. *Br. J. Cancer*, **51**, 681.

GABIZON, A. & PAPAHADJOPOULOS, D. (1988). Liposome formulations with prolonged circulation time in blood and enhanced uptake by tumors. *Proc. Natl Acad. Sci. USA*, **85**, 6949.

GABIZON, A., HUBERTY, J., STRAUBINGER, R.M., PRICE, D.C. & PAPAHADJOPOULOS, D. (1988). An improved method for *in vivo* tracing and imaging of liposomes using a Gallium 67-deferoxamine complex. *J. Liposome Res.*, **1**, 123.

GABIZON, A. (1989). Liposomes as a drug delivery system in cancer chemotherapy. In *Drug Carrier Systems*, Roerdink, F.H. & Kroon, A.M. (eds), p. 185. Wiley: Chichester.

GABIZON, A., PERETZ, T., SULKES, A. & 6 others (1989*a*). Systemic administration of doxorubicin-containing liposomes: a phase I study. *Eur. J. Cancer Clin. Oncol.*, **25**, 1795.

GABIZON, A., SHIOTA, R. & PAPAHADJOPOULOS, D. (1989*b*). Pharmacokinetics and tissue distribution of doxorubicin encapsulated in liposomes with long circulation times. *J. Natl Cancer Inst.*, **81**, 1484.

GOREN, D., GABIZON, A. & BARENHOLZ, Y. (1990). The influence of physical characteristics of liposomes containing doxorubicin on their pharmacological behaviour. *Biochim. Biophys. Acta*, **1029**, 285.

GREENE, R.F., COLLINS, J.M., JENKINS, J.F., SPEYER, J.L. & MYERS, C.E. (1983). Plasma pharmacokinetics of adriamycin and adriamycinol: implications for the design of *in vitro* experiments and treatment protocols. *Cancer Res.*, **43**, 3417.

HWANG, K.J. (1987). Liposome pharmacokinetics. In *Liposomes: From Biophysics to Therapeutics*, Ostro, M.J. (ed.), p. 109. Marcel Dekker: New York.

MACSWEEN, R.N., ANTHONY, P.P. & SCHEUER, P.J. (1979). *Pathology of the liver*. Churchill Livingstone: Edinburgh.

MAYHEW, E., RUSTUM, Y. & VAIL, W.J. (1983). Inhibition of liver metastases of M5076 tumor by liposome-entrapped adriamycin. *Cancer Drug Deliv.*, **1**, 43.

MIDDLEMAN, E., LUCE, J. & FREI, E. (1971). Clinical trials with adriamycin. *Cancer*, **28**, 844.

MOERLEIN, S.M. & WELCH, M.J. (1981). The chemistry of gallium and indium as related to radiopharmaceutical production. *Int. J. Nuc. Med. Biol.*, **8**, 227.

NOLTENIUS, H.W. (1981). *Manual of Oncology* . Urban & Schwarzenberg: Baltimore-Munich.

O'BRYAN, R.M., LUCE, J., TALLEY, R.W., GOTTLIEB, J.A., BAKER, L.H. & BONADONNA, G. (1973). Phase II evaluation of adriamycin in human neoplasia. *Cancer*, **32**, 1.

PEREZ-SOLER, R. (1989). Liposomes as carriers of antitumor agents: toward a clinical reality. *Cancer Treat. Rev.*, **16**, 67.

POSTE, G. (1983). Liposome targeting *in vivo*: problems and opportunities. *Biol. Cell*, **47**, 19.

RAHMAN, A., TREAT, J., ROH, J.-K. & 4 others (1990). A phase I clinical trial and pharmacokinetic evaluation of liposome-encapsulated doxorubicin. *J. Clin. Oncol.*, **8**, 1093.

RICHARDSON, V.J., RYMAN, B.E., JEWKES, R.F. & 4 others (1979). Tissue distribution and tumor localization of 99m-Technetium-labelled liposomes in cancer patients. *Br. J. Cancer*, **40**, 35.

ROWLAND, M. & TOZER, T.N. (1989). *Clinical Pharmacokinetics: Concepts and Applications*. Lea & Febiger: Philadelphia.

STORM, G., ROERDINK, F.H., STEERENBERG, P.A., DE JONG, W.H. & CROMMELIN, D.J. (1987). Influence of lipid composition on the antitumor activity exerted by doxorubicin-containing liposomes in a rat solid tumor model. *Cancer Res.*, **47**, 3366.

VAN DE VELDE, C.J. (1986). The staging of hepatic metastases arising from colorectal cancer. *Recent Results Cancer Res.*, **100**, 85.

WEINER, R.E., THAKUR, M.L., GOODMAN, M. & HOFFER, P.B. (1979). Relative stability of In-111 and Ga-67 desferrioxamine and human transferrin complexes. In *Radiopharmaceuticals II: Proceedings of the 2nd Int'l Symposium on Radiopharmaceuticals (Seattle, 1979)*, Sodd, V.J., Hoogland, D.R., Allen, D.R., Ice, R.D. & Sorensen, J.A. (eds), p. 331. The Society of Nuclear Medicine: New York.